# Nutritional Status of Children and Its Associated Factors in Selected Earthquake-Affected VDCs of Gorkha District, Nepal

**DOI:** 10.1155/2020/5849548

**Published:** 2020-07-05

**Authors:** Asmita Shrestha, Chet Kant Bhusal, Binjwala Shrestha, Kiran Dev Bhattarai

**Affiliations:** ^1^Department of Community Medicine and Public Health, Maharajgunj Medical Campus, Institute of Medicine, Tribhuvan University, Kathmandu, Nepal; ^2^Department of Community Medicine, Universal College of Medical Science and Teaching Hospital, Tribhuvan University, Bhairahawa, Rupandehi, Nepal

## Abstract

**Background:**

Malnutrition is a major public health problem and most enveloping cause of morbidity and mortality among children and adolescents throughout the world. This study was aimed at assessing the nutritional status and associated factors among 6-10-year-old children in selected earthquake-affected areas of Gorkha district, Nepal.

**Methods:**

A community-based cross-sectional study among 420 mothers having children of age groups 6-10 years (with anthropometric measurement among children) was conducted using a mixed method in selected earthquake-affected areas of Gorkha district, Nepal, from October 2015 to April 2016. Gorkha was selected purposively from 14 earthquake-affected districts. Two village development committees were selected randomly among 6 having severe impact. Randomly, 5 wards were selected from each of the 2 village development committees. As the sample was 420, 42 children were selected randomly from every ward.

**Result:**

Among the 420 children, 31.9% were underweight, 51.9% were stunted, and 2.9% were wasted after the earthquake. Children who were more prone to being underweight were the following: male children (RR = 1.34 95% CI: 1.01-1.78) and children from illiterate mothers (RR = 2.49, 95% CI: 1.85-3.36), illiterate fathers (RR = 1.73, 95% CI: 1.32-2.27), and homemaker mothers (RR = 0.28, 95% CI: 0.20-0.38); children whose families were using nonimproved sources of water (RR = 2.60, 95% CI: 1.07-6.60); and households having food insecurity (RR = 12.97, 95% CI: 3.29-51.18). Similarly, children of illiterate fathers (RR = 1.67, 95% CI: 1.41-1.97), children of illiterate mothers (RR = 2.32, 95% CI: 1.91-2.83), children of homemaker mothers (RR = 0.59, 95% CI: 0.49-0.70), children whose family were using treated water (RR = 0.32, 95% CI: 0.15-0.67), and children from food insecure households (RR = 10.52, CI: 4.05-27.33) were found to be stunted. After adjustment, children from households consuming nonimproved water were 6 times more likely (OR = 6.75; 95% CI: 1.59-28.62) to be wasted.

**Conclusion:**

Illiterate mothers, illiterate fathers, mothers engaged in occupation other than household work, and food insecure households were found to be independent predictors of underweight and stunting. Nonimproved source of drinking water was found to be independent predictors of wasting.

## 1. Introduction

Malnutrition, the most widespread cause of morbidity and mortality among children and adolescents [1, 2], is a major public health problem throughout the developing world predominantly in South Asia and Sub-Saharan Africa [3–5]. Malnutrition results from an unbalanced diet that does not contain all the necessary nutrients and/or inadequate or excessive consumption of nutrients. In addition, diseases that interfere with the body's ability to use the nutrients consumed can result in malnutrition [6]. The major causes of malnutrition in children are due to inadequate food intake, poor and unhygienic food quality, and severe and repeated infectious diseases [7–9]. A previous study indicates that malnourished children who survive would possibly suffer from recurring illness and faltering growth, with irreversible damage to their development and cognitive abilities [10]. Moreover, a child's nutritional future begins with the mother's nutritional status in adolescence and during pregnancy [11].

The nutritional status of school-aged children impacts their health, cognition, and subsequently their educational achievement [12]. Malnutrition is a public health issue affecting a large number of school-aged children influencing their health, growth and development, and school academic performance in developing countries and countries in transition [12, 13]. Malnourished school-aged children may live a compromised life in the matter of health [12]. More than 200 million school-aged children were stunted (short for their age) by year 2000, and the proportion of stunted school-aged children with impaired physical and mental development is expected to grow up to 1 billion by the year 2020 unless tangible action is undertaken [14].

Although progress has been made towards international goals for the eradication of hunger and malnutrition, considerable work is still required to achieve them and to respond to emerging public health nutrition challenges [14]. Globally, an estimated 101 million children under five years of age, or 16 percent, were underweight (i.e., weight-for-age below −2SD) in 2011 [15]. Moreover, wasting low weight-for-height still threatens the lives of 50 million children across the globe. In 2014, more than half of all stunted children under the age of 5 lived in Asia. The majority of children, 34.3 million under the age of 5, are suffering from wasting in Asia [16, 17].

The prevalence of underweight and thinness in Southeast Asia is 39 percent and 35 percent, respectively [1, 13]. Similarly, the degree of the malnutrition is very high in Nepal [18]. According to the Nepal Demographic and Health Survey (NDHS) 2016, 36 percent of children under the age of 5 are stunted, 10 percent are wasted, 27 percent are underweight, and 1 percent are overweight (heavy for their height) [19]. Likewise, a study from 2015 in Kaski of Nepal conducted among 5-10-year-old children revealed that 44.2 percent, 12.3 percent, and 35.4 percent were stunted, wasted, and underweight, respectively [20]. The same study also correlated underweight with family occupation (*χ*^2^ value = 15.679, *p* value = 0.047) and economic status of family (*χ*^2^ value = 15.464, *p* value < 0.001), whereas wasting was linked with education status of the mother (*χ*^2^ value = 10.691, *p* value = 0.014) [20].

A powerful earthquake of 7.8 magnitude racked Nepal on 25 April 2015. On 12th May 2015, a second major earthquake of 7.3 magnitude struck and caused further death and injury and heightened fears and tension among the affected population [21]. Out of 75 districts, 35 have been affected and 14 districts were severely affected. Gorkha is one of severely affected districts. During disaster, necessary things like food security and the water supply system were damaged partially or completely. Notably, food shortage can result in serious protein energy malnutrition and other deficiencies, which further add burden of disease and mortality [22, 23]. Although there are several literatures regarding the nutritional status of young and under 5 children, only few studies have considered school-going adolescents. Amongst these studies, very few have indicated the nutritional status of school-going adolescents after natural calamities such as earthquakes. After the earthquake, there is likelihood of disruption in food security which could degrade the nutritional status of the people, especially children and adolescents living in the affected area. Thus, to reduce the malnutrition, it is necessary to identify the factors affecting nutritional status. Hence, this study was aimed at assessing the nutritional status and associated factors among 6-10-year-old children in earthquake-affected village development committees (VDCs) of Gorkha district.

## 2. Materials and Methods

### 2.1. Study Design and Source of Population

A community-based cross-sectional study design was applied in two earthquake-affected VDCs (Manbu and Saurpani) of Gorkha district of Nepal (where severe impact had occurred). The purpose of this study was to assess the nutritional status and associated factors among 6-10-year-old children between October 2015 and April 2016. A mixed method (both quantitative as well as qualitative) was used for the study. A quantitative technique was used to assess nutritional status as well as associated factors of malnutrition while a qualitative technique was used to explore, triangulate, and collect detailed information related to water hygiene and sanitation, health, and food security in the postearthquake situation. Mothers having children of age group 6-10 years of selected VDCs were included in the study for taking the information regarding their children's nutritional status for both the quantitative and qualitative methods. Similarly, children of age group 6-10 years of selected VDCs were included for anthropometric measurement. Children whose family was residing in the sampling area less than one year were excluded. Also, a child was randomly selected using a lottery method when there was more than one child in one household.

### 2.2. Sample Size Determination and Sampling Technique

**Figure 1:**
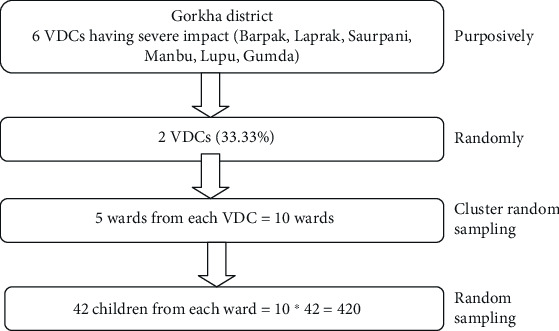
Sampling procedure.

The sample size of the study was 420 which was calculated by using the following formula: *N* = *Z*^2^*pq*/*L*^2^ [24] with 95% confidence interval, critical value *Z* = 1.96, absolute allowable error (L) = 6%, nonresponse rate = 2%, *p* = 0.26 (prevalence of undernourished children (26%) as found in the cross-sectional study done in Western Nepal [7], q = 1 − p = 1 − 0.26 = 0.74. Hence, *N* = (1.96)^2^∗(0.26)∗(0.74)/(0.06)^2^ = 205.31. As the cluster sampling technique was used, the initial sample size was multiplied by the design effect 2 which gives *N* = 205.31∗2 = 410.62. Considering a 2% nonresponse rate, the final sample size was 420. Similarly, two focus group discussions (FGDs) were conducted, one from each of the selected VDCs. The Gorkha district was selected purposively from 14 earthquake-affected districts as it was the worst earthquake-affected area. Similarly, Manbu and Saurpani which were severely impacted were selected randomly among 6 VDCs. From those two VDCs, each ward was taken as a cluster. So, 10 wards were taken randomly from the 2 VDCs with 5 wards from each VDC. As the total sample was 420, 42 6-10-year-old children were selected from each ward by random sampling (Figure 1).

### 2.3. Data Collection Procedure and Validity

Face-to-face interview was conducted with the mother of the child by using a structured questionnaire for collecting quantitative data. A separate focus group discussion (FGD) was conducted following the FGD guideline for qualitative data. Similarly, anthropometric measurements were carried out to assess the nutritional status of the children. The Nepal Demographic Healthy Survey (NDHS) [25] questionnaire was adapted for measuring the wealth index, household food insecurity, and household water and sanitation. The questionnaire was translated into Nepali, then into English and again into the Nepali language to find misapprehension and was pretested in Aruchanaute VDC of Gorkha district with 10 percent of the sample size, i.e., 42 samples. Two data collectors including the principal investigator with qualification of Master in Public Health and a trained enumerator with a Bachelor in Public Health were involved in data collection.

### 2.4. Data Processing and Analysis

Data coding, recoding, rechecking, and cleaning were continuously carried out to ensure data quality. Quantitative data was coded and entered in EpiData 3.1 version and analyzed by using SPSS 20 as well as the standard statistical software STATA version 13. Anthropometric calculations were done by using WHO AnthroPlus software [26]. Descriptive analysis was performed as per the study variables. In order to control clusters in the regression model, a robust standard error along with risk ratio was calculated. Since the prevalence of underweight and stunting was >10%, the risk ratio was calculated using a log-binomial regression model. Bivariate analysis was carried out to find the association between independent and dependent variables using the chi-square test for wasting. The variables, in bivariate analysis, that showed an association with the outcome variable with *p* value of 0.05 and less level of significance was further entered into the final multivariate logistic regression model after checking multicollinearity. The odds ratio along with the 95% confidence interval (CI) was estimated to assess the strength of the association and a *p* value < 0.05 was considered as statistically significant in the multivariable analysis. For qualitative data, all collected data was audio recorded along with notes taken by a trained enumerator. Immediately, it was transcribed in Nepali text by the researcher after each FGD was completed. They were then translated into English. Data analysis was carried out by coding, sorting, and summarizing the information into common themes. The data was analyzed as per the objectives of the study. The study has also enclosed italicized text which is supported by actual statements that were reported by the respondents, so as to represent their voices.

### 2.5. Setting

The Gorkha district is a part of the Gandaki Zone with Gorkha as its district headquarters. It is one of the worst earthquake-affected districts. It covers an area of 3610 km^2^ with 66,506 households. It has a total population of 271,061 with 121,041 males and 150,020 females. Manbu VDC has 1,388 households with a population of 6,016 and Saurpani VDC has 1,325 households with a population of 5,958 [27].

### 2.6. Anthropometric Measurement

The anthropometric measurements, following WHO guideline [28], were taken using the Seca digital weighing scale and Stature meter. The weighing scale was standardized daily with standard weights. The precision of the Seca digital scale was 0.1 kg. The height was recorded to the nearest 0.1 cm. Children were instructed to stand on the balance with light clothing and without footwear and with feet apart and looking straight. Height was taken without shoes and cap. Age of the child was calculated by subtracting date of birth from interviewed date. Mothers were asked to recall the date of birth using the local events calendar to determine the age in months if they forgot the actual date.

### 2.7. Operational Definition

#### 2.7.1. Nutritional Status

Nutritional status of child was measured in terms of weight for age (WAZ), height for age (HAZ), and weight for height (WHZ) as per WHO reference.

#### 2.7.2. Malnutrition

Malnutrition was determined by age and measurement of weight and height. It included underweight, stunting, and wasting.

#### 2.7.3. Underweight

Children whose weight for age was below minus two standard deviations (-2SD) from the median weight for age of the reference population were classified as underweight. It was dichotomized as underweight (if <-2SD) and not underweight (if >-2 SD).

#### 2.7.4. Stunting

Children whose height for age was below minus two standard deviations (-2SD) from the median height for age of the reference population were classified as stunted. It was dichotomized as stunted (if <-2SD) and not stunted (if >-2 SD).

#### 2.7.5. Wasting

Children whose weight for height was below minus two standard deviations (-2SD) from the median weight for height of the reference population were classified as wasted. It was dichotomized as wasted (if <-2SD) and not wasted (if >-2SD).

#### 2.7.6. Food Security

No food insecurity or experience of any of the three most severe conditions (running out of food, going to bed hungry, or going a whole day and night without eating).

*Mild food insecurity*: not having enough food sometimes or often; monotonous diet than desired but no cut back on quantity or experience of any of the three most severe conditions.

*Moderate food insecurity*: monotonous diet or undesirable foods sometimes or often; rarely or sometimes starting to cut back on quantity by reducing the size of meals or number of meals but do not experience any of the three most severe conditions.

*Severe food insecurity*: experienced one of these three conditions even once in the last 12 months.

#### 2.7.7. VDC Having Severe Impact

VDC which is severely affected by earthquake is listed by OSOCC (Government of Nepal, UNDAC, United Nations Agencies, nongovernmental organization and media sources).

#### 2.7.8. SLC

School leaving certificates.

## 3. Results

Among 420 mothers and their children enrolled in the study, more than half of children (57.4%) were 8-10 years old. More than half of the children (52.9%) were female. Regarding the ethnicity, two-thirds of the children were Janajatis followed by 18.6% Dalit, 12% Brahmin/Chhetri, and 2.1% Newar. Additionally, more than two-thirds were from nuclear family. More than half of the children (52%) were from the household having family members up to five. More than one-fourth of the children's fathers (28.1%) and more than two-fifths of the children's mothers (42.6%) were illiterate. Nearly one-third of both the children's fathers and mothers got informal education. Only 3% of children's fathers and 2.4% of children's mothers had completed school leaving certificate (SLC; 10^th^ grade level) and above level education. Regarding the occupation, nearly half of the children's fathers (44.5%) were involved in agriculture and nearly three-fifths of the children's mothers (59.5%) were involved in household chores (Table 1).

Majority of the households (96%) used improved water sources (e.g., piped water) for the purpose of drinking. Ninety-two percent of households did not treat drinking water. Majority of the households (88.8%) had a latrine facility. Among the households with latrines, majority, i.e., 84.5%, of the households were using a pit latrine. Regarding the handwashing practice of children, all the children washed hands before having meals and after defecation. However, only 42% children washed hands with soap (Table 2).

FGDs finding highlighted problems of clean drinking water and sanitation were most severe. Scarcity of water directly affects the sanitation as water is required for washing, cleaning, bathing and other purposes.

*Due to earthquake one toilet is shared by two households. One organization is helping in making toilet for household but community people are in dilemma that they have no house to live then how and where they can build toilet.* (FGD-Mothers from Saurpani)

FGD findings revealed that majority of mothers had knowledge about the nutritional values of the food. They reported that fruits, green vegetables, pulses, meat, fish, egg, and ghee (clarified butter) are nutritious food and these foods were occasionally incorporated in daily diet. However, the feeding practice of children was considerably changed after the earthquake. Also, economic problems heightened the difficulty in feeding nutritious food like meat, ghee, and fish. Notably, from the earthquake-affected people, a common concern was raised on the earthquake relief foods stating that the foods did not contain the required nutrients as compared to the local foods.

*There is also difficulty in availability of foods like meat, ghee, fish etc., as most of the animals are dead in the earthquake. We feed local rice before the earthquake but now we feed rice and other food stuffs that we got from relief. So, these foods did not contain the required amount of vitamins and nutrients.* (FGD-Mothers from Manbu)

Table 3 represents the household food security-related characteristics of the study population. The household food security includes availability, accessibility, and utilization of food within a year. More than four-fifths (84%) of households were food insecure where 23% of households were mildly food insecure, more than one-third (34.8%) were moderately food insecure, and slightly more than one-fourth (26%) were severely food insecure. Regarding the strategies adopted by households to cope with food insecurity, 85% of households with food insecurity took a loan to meet their food needs. Additionally, as the study area was earthquake affected, 92% of households relied on relief provided by different organizations and people to meet their food needs. All the respondents proclaimed that the earthquake was the major cause of their food insecurity. Seventy-five percent of households facing food insecurity reported financial problems (Table 3).

Furthermore, postearthquake drought also decreases the amount of crops harvested in comparison to previous years. In addition, the fragile condition of the crops forced the respondents to depend upon relief food. This scenario further led them to get loans and borrowing from relatives to fulfill their household needs, especially food. Mothers also shared their worries regarding the increase in the price of foodstuffs and other necessary goods in the market postearthquake.

*Only people whose houses were not damaged totally took out the stored crops from their house. Food stuffs from relief is enough only for small family but not for large family and the crops that we grew are only enough for 3-4* months*. So, we fulfill our need by doing daily labor and taking loans from others.* (FGD-Mothers from Saurpani)

This study highlights the nutritional status among 420 children surveyed. After a severe earthquake, nearly one-third (31.9%) of children were underweight, more than half (51.9%) were stunted, and 2.9% were wasted in the study area. The mean *Z*-score for weight for age (underweight) was -1.62 (95% CI: -1.692, -1.522), for height for age (stunting) was -2.12 (95% CI: -2.223, -2.017), and for weight for height (wasting) was -0.31 (95% CI: -0.388, -0.234) (Table 4).

Table 5 represents the log-binomial regression model of underweight to find the risk ratio with its associated factors. A chi-square test in bivariate analysis showed that sex of the child, education of the father, education of the mother, occupation of the mother, source of drinking water, and household food insecurity were significantly associated with underweight at 0.05 and less level of significance. The risk ratio of underweight was increased about 1.5 times (RR = 1.34, 95% CI: 1.01-1.78) for male children than for females. Children whose fathers were illiterate were 1.73 times (RR = 1.73, 95% CI: 1.32-2.27) at higher risk of having underweight in comparison to the children from literate fathers. The risk ratio of underweight was increased about 2.5 times (RR = 2.49, 95% CI: 1.85-3.36) for children of illiterate mothers than children of literate mothers. Similarly, the risk of being underweight decreased (RR = 0.28, 95% CI: 0.20-0.38) for children of homemaker mothers than for children of mothers other than homemakers. The risk of being underweight was increased 2.6 times (RR = 2.60, 95% CI: 1.07-6.60) for children whose family used water from nonimproved sources than for children from families who were using water from improved sources. In comparison to children from food secure households, the risk of being underweight for children from food insecure households increased around 13 times (RR = 12.97, 95% CI: 3.29-51.18) (Table 5).

Table 6 represents the log-binomial regression model of stunting to find the risk ratio with its associated factors. A chi-square test in bivariate analysis showed that education of the child's father, education of the child's mother, occupation of the child's mother, treatment of drinking water, and household food insecurity were significantly associated with stunting at 0.05 and less level of significance. The risk of being stunted increased nearly 2 times (RR = 1.67, 95% CI: 1.41-1.97) for children of illiterate fathers than for children of literate fathers. Similarly, the risk of being stunted increased 2.32 times (RR = 2.32, 95% CI: 1.91-2.83) for children of illiterate mothers than for children of literate mothers. The risks of being stunted decreased (RR = 0.59, 95% CI: 0.49-0.70) for children of homemaker mothers than for children of mothers other than homemakers. Children from the families who used treatment water were 0.32 times (RR = 0.32, 95% CI: 0.15-0.67) less likely to be stunted than the children from the families who were using water without treatment. The odds of being stunted increased nearly 10.5 times (RR = 10.52, CI: 4.05-27.33) for children from food insecure households than for children from food secure households (Table 6).

Table 7 represents the logistic regression analysis of wasting with its associated factors where type of family and source of drinking water were significantly associated with wasting at 0.05 and less level of significance in bivariate analysis. However, in the final logistic regression model, the independent predictor of increased odds for wasting was children from households who were consuming nonimproved sources of water (adjusted OR = 6.75, 95% CI: 1.59-28.62) (Table 7).

## 4. Discussion

The finding of this study revealed that 31.9% of the respondents were underweight, 51.9% were stunted, and 2.9% were wasted. Present findings were above the cutoff point from the significant level of public health, i.e., 30% for underweight and ≥40% for stunting, thus suggesting an immediate attention of all the concerned bodies such as VDCs, local NGOs/INGOs, and public health authorities [29]. The prevalence of wasting in our study from the Gorkha district was lower as compared to the study conducted in Kaski district [20]. Nonetheless, the prevalence of underweight and stunting was comparatively similar in both districts. Our methodology suggests a possible difference in study setting that could have brought up the disparity in the prevalence of wasting.

The prevalence of undernutrition particularly stunting was higher than underweight in the current study. A similar finding was also described in other researches that studied the prevalence of undernutrition [20, 30]. However, several studies from other countries [31–35] reported less prevalence of undernutrition in comparison to the present study. These differences on the prevalence of undernutrition might be due to the differential nutritional intake and socioeconomic and cultural differences rather than differences in their genetic potential to achieve maximum height. In the current study, education of the mother was significantly associated with underweight and stunting of children. This result is consistent with result of a study conducted in the western region of Nepal which reported that children of illiterate mothers were almost two times more likely to be malnourished than children of literate mothers [7]. Similarly, a study from Kaski district has shown that education of the mother was significantly associated with stunting of children [7, 20]. Also, studies from urban slums of India [33, 35] showed that education status of the mother was significantly associated with malnutrition of children.

The present study revealed that education of the father had significant association with the underweight and stunting of children; however, the study conducted in Central India reported a dissimilar result for underweight and stunting but a similar result with wasting [36]. This clearly emphasizes that educated mothers and fathers are aware of their child's nutrition, and the nutritional status of children directly depends on the mother's as well as father's education level. The present study also revealed that family size was not associated with underweight, stunting, and wasting of children. However, studies from other countries such as Egypt [37], Ethiopia [31, 32], and India [33, 35] reported that family size and the malnutrition of children have direct association. The possible cause of opposing views about the family size and children's malnutrition may be due to the disaster affecting the size of the family setting in our study. One of the strongest determinants of stunting may be the mother's occupation. In this study, children of homemaker mothers were less likely to be stunted than children of mothers who were engaged in other occupations. In support, the study conducted in Ethiopia [32] aligns with our notion highlighting that children from employed mothers were at greater risk of stunting than those from housewife mothers. Undoubtedly, these findings assert that a mother's care plays a major role in the child's nutrition as she is the closest to the child. Moreover, mothers who stayed at home could spend more time caring for their children. Wealth is a well-known factor that influences the nutritional status of the children. However, the current study noted that the wealth quintile was not associated with underweight, stunting, and wasting of children. A similar finding was also reported from the study conducted in Central India [36]. On the contrary, a separate research done in India showed that children from low-income households were nearly 3 times more likely to be underweight as well as stunted when compared to the children from high-income households [30]. In accord, a study from Mysore, India, showed that the low socioeconomic status was highly associated with underweight [38]. The discrepancy about the wealth associating with the child's nutritional status, in our study compared to other studies, could be the mass destruction of household assets and the chaotic situation caused by the 2015 earthquake. This study revealed that children of food insecure households are more likely to be underweight and stunted which is supported by a study done by Wolde and his colleagues [39]. This indicated that malnourished children from food insecure households are more prone to the food insufficiency created by the earthquake. Besides nutritional food, source of drinking water is an important factor that influences the health of children. In a bivariate analysis, children who belonged to households that used nonimproved sources like river/pond/stream for drinking were more likely to be underweight in comparison to children who belonged to households that used improved sources that include piped water. Furthermore, households that treated drinking water (filtration) were negatively associated with stunting of the children in the bivariate analysis. Indeed, the use of drinking water from nonimproved sources can cause the children to suffer from waterborne diseases. This condition may compromise the immunity of the child which can result in being underweight. Nonetheless, the incidence of waterborne diseases can be reduced with the proper treatment of drinking water.

## 5. Conclusion

The study revealed that undernutrition is prevalent in the 6-10-year-old school-aged children. Illiterate mothers, illiterate fathers, mothers engaged in occupations other than household work, and food insecure households were found to be independent predictors of increased risks for underweight and stunting. Nonimproved sources of drinking water were found to be independent predictors of wasting. On the other hand, wealth quintile, family type, family size, and occupation of the father were not significantly associated with all the three indicators of malnutrition in this study. None of the sociodemographic variables were significantly associated with wasting of children. Still, natural calamities such as an earthquake intensify the severity of the malnutrition in children due to the loss of crops, lack of clean drinkable water, proper hygiene, and sanitation. Based on these findings, efforts should be done to promote the education of women and men by providing informal learning opportunity, and intervention addressing the problems of household food insecurity should be emphasized.

## Figures and Tables

**Table 1:** Background related characteristics of study population.

Characteristics	Number (*n* = 420)	Percentage
Age of the child		
6-7 years	179	42.6
8-10 years	241	57.4
Sex		
Male	198	47.1
Female	222	52.9
Education of child's father		
Illiterate	118	28.1
Informal education	131	31.2
Primary level	97	23.1
Secondary level	61	14.5
SLC and above	13	3.1
Education of child's mother		
Illiterate	179	42.6
Informal education	132	31.4
Primary level	56	13.3
Secondary level	43	10.2
SLC and above	10	2.4
Occupation of child' s father		
Service	38	9.0
Agriculture	187	44.5
Business	3	0.7
Foreign employee	65	15.5
Daily labor	114	27.1
Others	13	3.1
Occupation of child' s mother		
Homemaker	250	59.5
Agriculture	150	35.7
Service	9	2.1
Daily labor	6	1.4
Business	2	0.5
Others	3	0.7

**Table 2:** Household water- and sanitation-related characteristics of the study population.

Characteristics	Number (*n* = 420)	Percentage
Main source of drinking water		
Improved source		
Piped water	402	95.7
Nonimproved source		
River/pond/stream	18	4.3
Water treatment prior to drinking		
Yes	35	8.3
No	385	91.7
Latrine in household		
Yes	373	88.8
No	47	11.2
Type of latrine (*n* = 373)		
Flush	18	4.3
Pit latrine	355	84.5
Handwashing practice		
Before having meal	420	100.0
After having meal	402	95.7
After defecation	418	99.5
Others	3	0.7
Handwashing materials		
Water only	241	57.4
Soap	175	41.7
Ash/mud	4	1.0

**Table 3:** Household food insecurity-related characteristics of study population.

Characteristics	Number (*n* = 420)	Percent
Food security		
Food security	69	16.4
Mild food insecurity	96	22.9
Moderate food insecurity	146	34.8
Severe food insecurity	109	26.0
Coping strategies of food insecurity		
Take loan	350	84.5
Collect wild food	2	0.5
Consume seed	20	4.8
Sell livestock	1	0.2
Relief	381	92.0
Cause of food deficiency		
Earthquake	414	99.8
Drought	27	6.5
Crop failure	27	6.5
Financial problem	309	74.5
Not available in the market	7	1.7
Others	1	0.2

**Table 4:** Prevalence of nutritional status of children.

Characteristics	Number	Percentage
Underweight	134	31.9
Mean *Z*-score for weight for age (95% CI)	-1.61 (-1.692, -1.522)	
Stunting	218	51.9
Mean *Z*-score for height for age (95% CI)	-2.12 (-2.223, -2.017)	
Wasting	12	2.9
Mean *Z*-score for weight for height (95% CI)	-0.31 (-0.388, -0.234)	

**Table 5:** Factors associated with underweight in using log-binomial regression model.

Characteristics	Underweight status (%)		*p* value	Robust standard error	RR 95% CI
	Underweight	Normal weight			
Sex					
Male	73 (36.9)	125 (63.1)	0.039^∗^	0.034	1.34 (1.01-1.78)
Female	61 (27.5)	161 (72.5)			1
Family size					
>5 persons	70 (35.0)	130 (65.0)	0.194	0.034	1.14 (0.93-1.41)
≤5 persons	64 (29.1)	156 (70.9)			1
Education of father					
Illiterate	54 (45.8)	64 (54.2)	0.001^∗^	0.037	1.73 (1.32-2.27)
Literate	80 (26.5)	222 (73.5)			1
Education of mother					
Illiterate	87 (48.6)	92 (51.4)	<0.001^∗^	0.033	2.49 (1.85-3.36)
Literate	47 (19.5)	194 (80.5)			1
Occupation of mother					
Homemaker	39 (15.6)	211 (84.4)	<0.001^∗^	0.031	0.28 (0.20-0.38)
Other than homemaker	95 (55.9)	75 (44.1)			1
Source of drinking water					
Nonimproved source	10 (55.6)	8 (44.4)	0.028^∗^	0.026	2.60 (1.07-6.60)
Improved source	124 (30.8)	278 (69.2)			1
Treatment of drinking water					
Yes	6 (17.1)	29 (82.9)	0.50	0.026	0.44 (0.18-1.03)
No	128 (33.2)	257 (66.8)			1
Household food insecurity					
Yes	132 (37.6)	219 (62.4)	<0.001^∗^	0.027	12.97 (3.29-51.18)
No	2 (2.9)	67 (97.1)			1

^*^Significant at *p* < 0.05. 1: reference category; RR: risk ratio.

**Table 6:** Factors associated with stunting in using log-binomial regression model.

Characteristics	Stunting status (%)		*p* value	Robust standard error	RR 95% CI
	Stunted	Normal			
Education of father					
Illiterate	86 (72.9)	32 (27.1)	<0.001^∗^	0.031	1.67 (1.41-1.97)
Literate	132 (43.7)	170 (56.3)			1
Education of mother					
Illiterate	138 (77.1)	41 (22.9)	<0.001^∗^	0.029	2.32 (1.91-2.83)
Literate	80 (33.2)	161 (66.8)			1
Occupation of mother					
Homemaker	101 (40.4)	149 (59.6)	<0.001^∗^	0.030	0.59 (0.49-0.70)
Other than homemaker	117 (68.8)	53 (31.2)			1
Treatment of drinking water					
Yes	9 (25.7)	26 (74.3)	0.001^∗^	0.061	0.32 (0.15-0.67)
No	209 (54.3)	176 (45.7)			1
Latrine in household					
Yes	188 (50.4)	185 (49.6)	0.083	0.046	0.94 (0.88-1.01)
No	30 (63.8)	17 (36.2)			1
Household food insecurity					
Yes	214 (61.0)	137 (39.0)	<0.001^∗^	0.031	10.52 (4.05-27.33)
No	4 (5.8)	65 (94.2)			1

^*^Significant at *p* < 0.05. 1: reference category; RR: risk ratio.

**Table 7:** Factors associated with wasting in using bivariate and multivariate analyses.

Characteristics	Wasting status (%)		*p* value	COR^a^ 95% CI	AOR^b^ 95% CI
	Wasted	Normal			
Sex					
Male	8 (4.0)	190 (96.0)	0.280	2.30 (0.68-7.74)	—
Female	4 (1.8)	218 (98.2)		1	
Family type					
Joint/extended	7 (5.6)	117 (94.4)	0.026	3.48 (1.08-11.19)	2.89 (0.87-9.58)
Nuclear	5 (1.7)	291 (98.3)		1	1
Family size					
>5 person	8 (4.0)	192 (96.0)	0.295	2.25 (0.67-7.59)	—
≤5 person	4 (1.8)	216 (98.2)		1	
Occupation of child's father					
Agriculture	3 (1.6)	184 (98.4)	0.167	0.41 (0.11-1.52)	—
Other than agriculture	9 (3.9)	224 (96.1)		1	
Source of drinking water					
Nonimproved source	3 (16.7)	15 (83.3)	0.004^∗^	8.73 (2.14-35.58)	6.75 (1.59-28.62)
Improved source	9 (2.2)	393 (97.8)		1	

^∗^Significant at *p* < 0.05. 1: reference category; ^a^crude odds ratio, ^b^adjusted odds ratio.

## Data Availability

The raw data under identification policy will be provided, upon request through email to any of the corresponding authors.

## Abbreviations

AOR: Adjusted odds ratio
CI: Confidence interval
COR: Crude odds ratio
FGD: Focus group discussion
GSHS: Global school-based student health survey
NDHS: Nepal Demographic and Health Survey
OR: Odds ratio
RR: Risk ratio
RSE: Robust standard error
SBA: Skilled birth attendant
SD: Standard deviation
SEAR: Southeast Asia region
SLC: School leaving certificate
SPSS: Statistical Package for the Social Sciences
VDC: Village development committee
WHO: World Health Organization.
